# Factors Associated With the Occurrence and Evolution of Recent Small Subcortical Infarcts (RSSIs) in Different Locations

**DOI:** 10.3389/fnagi.2020.00264

**Published:** 2020-08-26

**Authors:** Hui Hong, Ruiting Zhang, Xinfeng Yu, Yeerfan Jiaerken, Shuyue Wang, Xiao Luo, Min Lou, Peiyu Huang, Minming Zhang

**Affiliations:** ^1^Department of Radiology, The Second Affiliated Hospital, School of Medicine, Zhejiang University, Hangzhou, China; ^2^Department of Neurology, The Second Affiliated Hospital, School of Medicine, Zhejiang University, Hangzhou, China

**Keywords:** recent small subcortical infarcts, subcortical white matter, basal ganglia region, brainstem, white matter hyperintensities

## Abstract

Recent small subcortical infarcts (RSSIs) can occur in different brain regions. Distinct etiologies might be involved for RSSIs in different locations and could further affect RSSI cavitation and functional outcomes. In this study, we aim to analyze the baseline clinical and imaging characteristics associated with the occurrence and cavitation of RSSIs in different locations. We retrospectively include patients who presented with RSSIs from a database for cerebral small vessel disease. Detailed information, including demographic, clinical, laboratory, and radiological data, were collected. We identify baseline RSSIs on diffusion-weighted images and divide them into brainstem, subcortical white matter, and basal ganglia region groups. Cavitation is evaluated on follow-up T2 fluid-attenuated inversion recovery (FLAIR) images. Statistical analysis is performed to determine factors associated with the occurrence and cavitation of RSSIs in different locations. We find that patients with brainstem RSSIs have a higher proportion of diabetes (64.1%) compared to patients with subcortical white matter (27.3%, *P* < 0.001) and basal ganglia region RSSIs (35.2%, *P* = 0.006) and have higher levels of HbA1c (7.20%) compared to patients with subcortical white matter (6.10%, *P* = 0.001) and basal ganglia region RSSIs (6.20%, *P* = 0.003). In addition, patients with brainstem RSSIs have higher NIHSS scores than patients with subcortical white matter RSSIs (2 vs 0, *P* = 0.001). Patients with subcortical white matter RSSIs have higher a white matter hyperintensity (WMH) burden compared to patients with basal ganglia region RSSIs (21.64 cm^3^ vs 11.10 cm^3^, *P* = 0.004). Follow-up analysis demonstrates that basal ganglia region RSSIs are less likely to cavitate than subcortical white matter RSSIs (61.4% vs 83.6%, *P* = 0.010), and contacting with WMH is associated with the cavitation of subcortical white matter RSSIs (OR: 101.760, *P* = 0.003). Our study demonstrates that RSSIs in different locations are associated with different clinical and imaging characteristics. Furthermore, cavitation of RSSIs might be affected by local lesion features and the surrounding environment rather than general demographic and clinical factors.

## Introduction

Recent small subcortical infarcts (RSSIs) are defined as recent infarctions in the territory of a perforating arteriole with consistent imaging features or clinical symptoms in the previous few weeks (Caplan, 2015), accounting for about 25% of ischemic strokes (Caplan, 2015; Regenhardt et al., 2018). RSSIs can occur in three locations, including the brainstem, subcortical white matter, and basal ganglia region. Previous studies find that RSSIs in different locations could have different etiologies (Nah et al., 2010; Kloppenborg et al., 2017; Eppinger et al., 2019), and their association with vascular risk factors and concomitant cerebrovascular conditions also varies (Gouw et al., 2008; Del Bene et al., 2013).

The evolution of RSSIs has significant clinical meanings. RSSIs can evolve into either cavities or non-cavitated lesions (Potter et al., 2010; Koch et al., 2011). Cavitated RSSIs, i.e., lacunes, represent severe loss of neuronal tissues and are associated with poorer clinical outcomes (Pinter et al., 2018; Duering et al., 2019) compared to non-cavitated RSSIs. Furthermore, the contribution of lacunes to clinical impairments depends on their numbers and locations (Van Der Flier et al., 2005; Benjamin et al., 2014; Pavlovic et al., 2014). One single strategic lacune can cause severe symptoms. Therefore, investigating factors associated with the cavitation of RSSIs in different brain regions may provide important evidence for future clinical management (Benjamin et al., 2014; Biesbroek et al., 2017).

Several previous studies find that the evolution of RSSIs is relatively stable after 3 months, and their cavitation is associated with lesion size, follow-up time, and preexisting white matter hyperintensities (WMH) (Koch et al., 2011; Okazaki et al., 2015; Loos et al., 2018; Pinter et al., 2018; Duering et al., 2019). Two studies demonstrate that the cavitation rate of RSSIs in different locations might be distinct. One has very small sample size (Moreau et al., 2012), and the other one (PICASSO substudy) (Kwon et al., 2019) concludes that RSSIs located in the anterior circulation regions are more likely to cavitate. However, as patients in the PICASSO substudy, all had a history of intracerebral hemorrhage or the presence of multiple cerebral microbleeds, so the conclusions may not be applicable to general populations with RSSIs.

Therefore, we designed this study with the following objectives: (1) to analyze the baseline clinical and imaging characteristics of RSSIs in different locations, and (2) to assess their evolution pattern and discover the factors affecting RSSI cavitation in different locations.

## Materials and Methods

### Study Population and Recruitment

**FIGURE 1:**
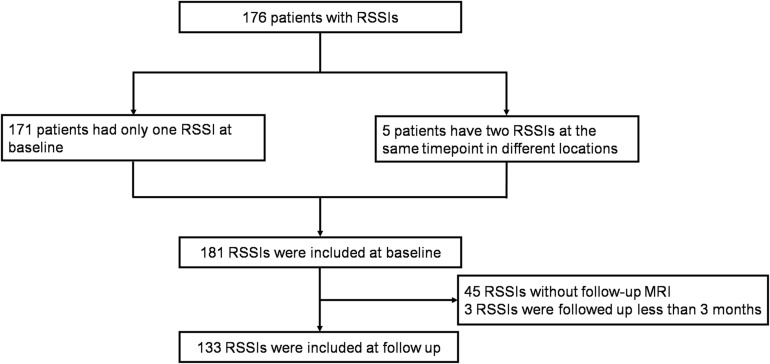
Inclusion flow chart. One hundred seventy-six patients with RSSIs were included at baseline and RSSIs with follow-up MRI more than 3 months were included at follow-up.

We retrospectively reviewed our prospectively collected database, consisting of cerebral small vessel disease (CSVD) patients (defined as the presence of lacunes and/or WMH on MRI) between July 30, 2012 and August 26, 2016. We included patients with identified RSSIs on diffusion-weighted imaging (DWI). Exclusion criteria were (1) any cortical ischemic lesion and (2) any other imaging evidence of preexisting structural brain lesion (hemorrhage, cerebral trauma, cerebrovascular malformation, and brain tumors). In the follow-up analysis, we excluded patients who were scanned within fewer than 3 months (Okazaki et al., 2015) because the status of RSSIs is not stable within 3 months. Please see the study flowchart (Figure 1).

Demographics, vascular risk factors, imaging characteristics, and laboratory data as well as the National Institutes of Health Stroke Scale (NIHSS) scores were collected. We extracted information on the vascular risk factors from the electronic medical documentation system of our hospital. Vascular risk factors were defined as arterial hypertension (preexisting diagnosis or blood pressure ≥140/90 mmHg), hypercholesterolemia (preexisting diagnosis or total cholesterol ≥5.72 mmol/L or TG ≥ 1.70 mmol/L.), diabetes mellitus (preexisting diagnosis or fasting blood glucose ≥7.0 mmol/L or 2-h postprandial blood glucose ≥11.1 mmol/L or glycated hemoglobin (HbA1c) ≥6.5%), and cardiac disease [including atrial fibrillation, coronary heart disease, and cardiac hypertrophy, which was based on preexisting diagnosis or diagnosis during hospitalization according to echocardiography, electrocardiogram (ECG) examination, and coronary artery, CT]. Laboratory data included the first blood pressure on admission, fasting blood glucose, HbA1c, and C-reaction protein (CRP) from the next morning upon the patient’s arrival.

### Standard Protocol Approvals, Registrations, and Patient Consents

Study protocols were approved by the local ethics committee. All clinical investigation was conducted according to the principles expressed in the Declaration of Helsinki. All participants signed informed consent on admission.

### Image Acquisition

Baseline MR images were acquired on 1.5 T scanners (Siemens/Gemedical System), including Axial 2D T1-weighted sequence [repetition time (TR) = 2,060 ms; echo time (TE) = 25.2 ms; inversion time (TI) = 720 ms; matrix = 320 × 224; voxel size = 0.75 × 1.07 × 6 mm^3^; flip angle = 90°; slice thickness = 6 mm], axial T2 fluid-attenuated inversion recovery (FLAIR) sequence (TR = 9,002 ms; TE = 152 ms; TI = 2,100 ms; 256 × 192 matrix; voxel size = 0.94 × 1.25 × 5 mm^3^; flip angle = 90°; slice thickness = 5 mm), and axial DWI (TR = 4,200 ms; TE = 82 ms; 128 × 128 matrix; voxel size = 1.88 × 1.88 × 6/7 mm^3^; slice thickness = 6–7 mm; b = 1,000 s/mm^2^) with apparent diffusion coefficient (ADC) maps.

Follow-up MR imaging was acquired on a 3 T scanner (General Electric Medical System, Discovery MR 750), including axial FLAIR (TR = 8,400 ms; TE = 150 ms; IR = 2,100 ms; 256 × 256 matrix; voxel size = 0.47 × 0.47 × 4 mm^3^; slice thickness = 4 mm) and axial DWI (TR = 5,000 ms; TE = 86 ms; 160 × 160 matrix; voxel size = 0.94 × 0.94 × 4 mm^3^; flip angle = 90°; slice thickness = 4 mm, b = 1,000 s/mm^2^).

### Evaluation of RSSIs

**FIGURE 2:**
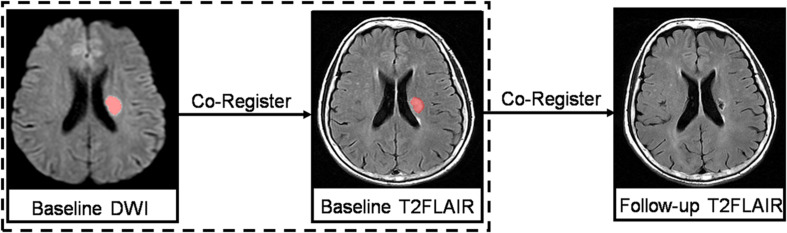
Baseline DWIs were registered to the baseline and follow-up T2 FLAIR images to ensure observation of the same lesion.

**FIGURE 3:**
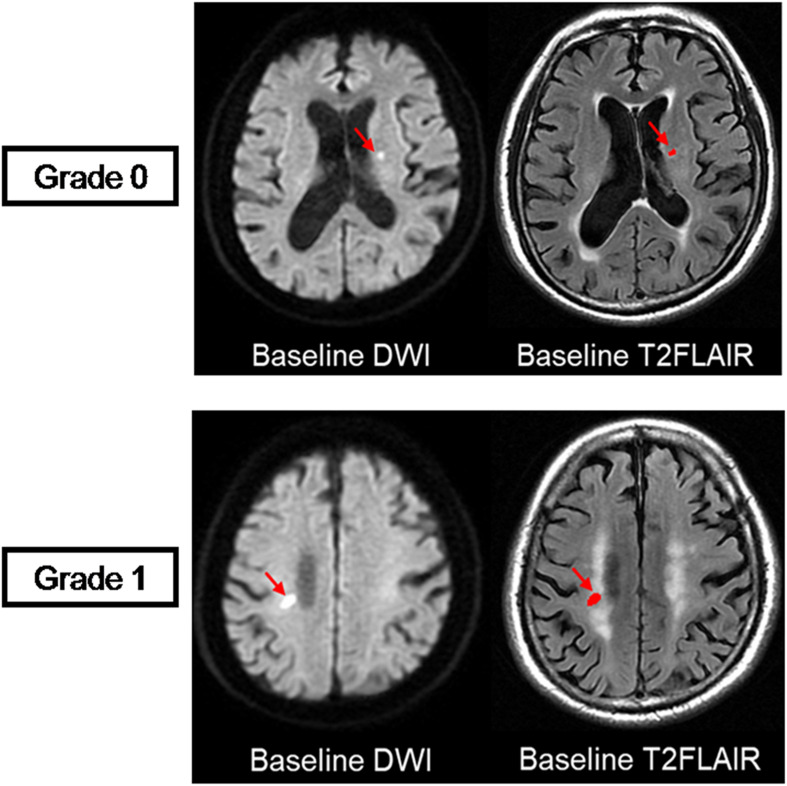
Visual rating scale. Spatial relationship between RSSIs and surrounding white matter hyperintensities are rated at baseline. The top row shows an RSSI on baseline T2 FLAIR (red arrow) isolated from the surrounding WMH, which is rated as grade 0. The bottom row shows an RSSI on baseline T2 FLAIR (red arrow) contacting with the surrounding WMH, which is rated as grade 1.

Recent small subcortical infarcts were defined as subcortical hyperintensity lesions on DWI with maximal diameter ≤25 mm (by axial) that are located in the blood-supply area of a small penetrating artery. RSSIs were manually segmented with MRIcron software^1^ (University of South Carolina, Columbia, SC, United States) on DWI and then coregistered to baseline T2 FLAIR images and follow-up T2 FLAIR images to confirm the observation of the same lesion (Figure 2). The location (brainstem; basal ganglia region, which included basal ganglia nucleus and thalamus; and subcortical white matter) of each RSSI was recorded. The longest diameter of each subcortical infarct was measured in the axial planes on DWI. In addition, we assessed their spatial relationship with the surrounding WMH (RSSIs contacting surrounding WMH were rated as grade 1; otherwise, they were rated as grade 0; Figure 3).

**FIGURE 4:**
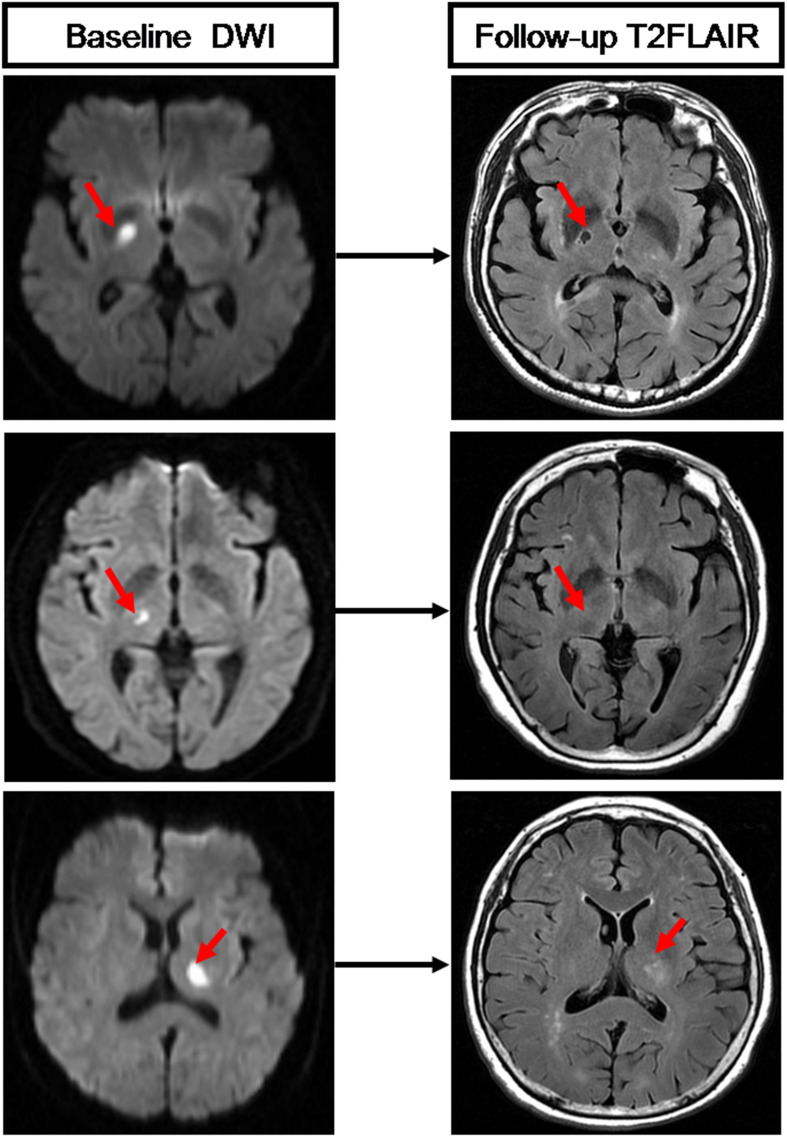
Three RSSIs show different evolution patterns. The top row shows an RSSI (red arrow) has evolved into a lacune on follow-up T2 FLAIR. The medium row shows an RSSI (red arrow) has disappeared on follow-up T2 FLAIR. The bottom row shows an RSSI (red arrow) that remained as white matter hyperintensities on follow-up T2 FLAIR.

On follow-up MRI scans, T2 FLAIR images were used to evaluate the cavitation of RSSIs. A cavity was defined as a lesion with consistent CSF signal intensity (hypointensity) surrounded by a high signal circle on T2 FLAIR. RSSIs that evolved into hyperintensity lesions as WMH or disappeared on follow-up T2 FLAIR images were considered as non-cavitation (Figure 4). Two neuroradiologists (HH and RZ), who were blinded to all other clinical and imaging data of the patients, viewed the follow-up T2 FLAIR images and assessed RSSI cavitation independently. The interobserver κ was 0.824 [95% confidence interval (CI): 0.70–0.97]. Discrepancies were resolved by consensus. A neuroradiologist (HH) performed the cavitation assessment twice at an interval of 3 months. The intraobserver κ was 0.842 (95% CI: 0.70–0.97).

### Measurement of WMH

T2 FLAIR images were used for WMH lesion segmentation. We utilized an automatic segmentation tool (Lesion Segmentation Tool, Version 2.0.15), which was based on SPM 12 (Statistical Parametric Mapping), using a lesion prediction algorithm (LPA) that requires only FLAIR images. The automatically created WMH label images were then manually corrected for scalp and tissue falsely classified as WMH and WMH falsely classified as normal white matter. Volumes of segmented WMH were extracted with the “Extract values of interest” function in LST.

### Statistical Analysis

Demographic, imaging, and clinical data were analyzed with the Statistical Package of Social Science (IBM SPSS Statistics 23). We used the Kolmogorov–Smirnov test to assess normality of data distribution. Baseline characteristics were compared among the three groups, using chi-square test (for categorical data) and Mann–Whitney *U* test (for non-normally continuous variables). Multiple comparison correction was carried out by applying Bonferroni correction for three locations, and *p* < 0.017 (0.05/3) was considered significant.

To determine factors associated with cavitation, univariate analysis was first performed for RSSIs in different locations to identify relevant factors. Variables with *p* < 0.1 in univariate analyses were included in the multiple logistic regression model. All analyses were performed blinded to the participant-identifying information.

## Results

During the study period (between July 30, 2012 and August 26, 2016), 176 CSVD patients with RSSIs were included. Among them, five patients had more than one RSSI at the same time point in different locations. Therefore, 181 RSSIs were analyzed at baseline. In the follow-up study, we excluded 45 patients who didn’t come to our hospital for reexamination and three patients who were followed up within less than 3 months. Finally, 133 RSSIs were analyzed for cavitation. Comparison of clinical data and MRI findings of RSSIs with and without follow-up MRI is shown in Supplementary Table S1.

Briefly, the patients’ median age was 68 (63–77) years, and 111 (61.3%) of them were men. The median NIHSS score was 1 (0–2), and the median time of the initial MRI to symptom onset was 1.06 (1–3) days. Thirty-nine (21.5%) RSSIs were in the brainstem, 88 (48.6%) were in subcortical white matter, and 54 (29.9%) were in the basal ganglia region.

### Cross-Sectional Analysis of RSSIs of Different Locations

Patients with brainstem RSSIs had a higher proportion of diabetes compared to patients with subcortical white matter and basal ganglia region RSSIs (64.1% vs 27.3% vs 35.2%, *P* < 0.001, *P* = 0.006) and had higher levels of HbA1c compared to patients with subcortical white matter and basal ganglia region RSSIs (7.20% vs 6.10% vs 6.20%, *P* = 0.001, *P* = 0.003). Brainstem RSSIs were larger than subcortical white matter RSSIs (11.70 mm vs 9.27 mm, *P* = 0.005), and patients with brainstem RSSIs had higher NIHSS scores than patients with subcortical white matter RSSIs (2 vs 0, *P* = 0.001). Patients with subcortical white matter RSSIs had a higher WMH burden compared to patients with basal ganglia region RSSIs (21.64 cm^3^ vs 11.10 cm^3^, *P* = 0.004). Additionally, subcortical white matter RSSIs were more frequently contacted with surrounding WMH compared with brainstem RSSIs and basal ganglia region RSSIs (67.0% vs 2.6% vs 11.1%, *P* < 0.001, *P* < 0.001). Detailed characteristics of RSSIs in different locations at baseline are shown in Table 1.

**TABLE 1:** Clinical data and MRI findings of baseline RSSIs according to location.

**Location**	**Total (*n* = 181)**	**Basal ganglia region (*n* = 54)**	**Brainstem (*n* = 39)**	**Subcortical white matter (*n* = 88)**
**Demographic characteristics**				
Age, y (IQR)	68 (63–77)	68 (63–75)	66 (61–77)	69 (63–78)
Male, *n* (%)	111 (61.3%)	31 (57.4%)	29 (74.4%)	51(58.0%)
^†^Time of the initial MRI to symptom onset, d (IQR)	1.06 (1–3)	1.33 (0.85–3.00)	1.54 (1–4)	1 (1–2.75)
NIHSS, score (IQR)	1 (0–2)	**1 (0–2)^*a,b*^**	**2 (0–4)^*a*^**	**0 (0–2)^*b*^**
**Vascular risk factors**				
Diabetes, *n* (%)	68 (37.6%)	**19 (35.2%)^*a*^**	**25 (64.1%)^*b*^**	**24 (27.3%)^*a*^**
Hypertension, *n* (%)	148 (81.8%)	44 (81.5%)	35 (89.7%)	69 (78.4%)
Hyperlipemia, *n* (%)	28 (15.5%)	8 (14.8%)	7 (17.9%)	13 (14.8%)
Cardiac disease, *n* (%)	19 (10.5%)	4 (7.4%)	1 (2.6%)	14 (15.9%)
**Clinical index**				
CRP, mmol/L (IQR)	1.90 (0.80–5.10)	2.00 (0.92–5.90)	1.90 (0.80–8.53)	1.60 (0.60–4.00)
HbA1c,% (IQR)	6.30 (5.80–7.20)	**6.20 (5.70–7.00)^*a*^**	**7.20 (6.20–9.35)^*b*^**	**6.10 (5.80–6.93^*a*^**
Glu, mg/L (IQR)	5.28 (4.73–6.49)	5.34 (4.76–6.45)	5.50 (4.58–8.00)	5.26 (4.68–6.18)
Systolic pressure, mmHg (IQR)	152 (140–166)	155 (141–167)	156 (144–166)	149 (136–160)
Diastolic blood pressure, mmHg (IQR)	85 (76–94)	88 (76–96)	84 (74–94)	84 (76–94)
**Imaging characteristics**				
ADC × 10^–6^, mm^2^/s (IQR)	559 (492–663)	553 (500–626)	567 (474–690)	577 (491–671)
RSSIs diameter, mm (IQR)	9.60 (7.62–12.95)	**9.45 (7.98–12.50)^*a,b*^**	**11.70 (7.92–15.00)^*a*^**	**9.27 (6.95–11.60)^*b*^**
^#^Total WMH volume, cm^3^	15.15 (7.37–33.21)	**11.10 (4.51–24.41)^*a*^**	**11.52 (7.37–26.78)^*a,b*^**	**21.64 (10.20–36.39)^*b*^**
Contact with surrounding WMH, *n* (%)	66 (36.5%)	**6 (11.1%)^*a*^**	**1 (2.6%)^*a*^**	**59 (67.0%)^*b*^**
^‡^Cavity formation on follow-up MRI	101 (75.9%)	**51 (83.6%)^*a*^**	**23 (82.1%)^*a,b*^**	**27 (61.4%)^*b*^**

*Data are presented as median (IQR) or number (%). CRP, c-reactive protein; ADC, apparent diffusion coefficient; RSSIs, recent small subcortical infarcts; WMH, white matter hyperintensities. Bold numbers represent the significant statistical difference (*p* < 0.05) among three locations. *Post hoc* results are demonstrated by superscripts a/b. Two groups with different superscripts have significant statistical difference (*p* < 0.05/3) in *post hoc* multiple comparisons, while groups with the same superscripts had no significant difference from each other. ^†^13 lesions were found by coincidence without definite time of symptom onset. ^#^Analysis of total WMH volume was performed in 162 patients (five patients had two RSSIs at the same time point), and 14 patients didn’t have baseline T2 FLAIR images. ^‡^Analysis of cavity formation on follow-up MRI were performed in 133 RSSIs (48 baseline RSSIs without follow up MRI images).*

### Longitudinal Analysis of RSSIs of Different Locations

Our follow-up study shows that basal ganglia region RSSIs are less likely to cavitate compared to subcortical white matter RSSIs (61.4% vs 83.6%, *P* = 0.010).

Details of the univariate analysis on RSSI cavitation are shown in Table 2. In multivariate logistic regression, we find that, for RSSIs in the basal ganglia region, larger baseline lesion diameter is independently associated with RSSI cavitation (OR: 1.355, 95% CI: 1.009–1.822, *P* = 0.044) although, for subcortical white matter RSSIs, contacting with surrounding WMH (OR: 101.760, 95% CI: 4.717–2195.290, *P* = 0.003) is independently associated with cavitation (Tables 3, 4). As only age was associated with cavitation in the brainstem (*P* = 0.050), we did not perform multiple regression analyses for this region.

**TABLE 2:** Comparisons of RSSIs in different locations with and without cavity formation on follow-up MRI.

**Location**	**Basal ganglia region**			**Brainstem**			**Subcortical white matter**		
	**Cavitation (*n* = 27)**	**Non-cavitation (*n* = 17)**	***P* Value**	**Cavitation (*n* = 23)**	**Non-cavitation (*n* = 5)**	***P* Value**	**Cavitation (*n* = 51)**	**Non-cavitation (*n* = 10)**	***P* Value**
**Demographic characteristics**									
Age, y (IQR)	69 (64–77)	68 (58–73)	0.538	65 (59–72)	77 (68–79)	**0.050**	70 (64–79)	67 (60–69)	0.141
Male, *n* (%)	16 (59.3%)	10 (58.8%)	1.000	15 (65.2%)	4 (80.0%)	1.000	29 (56.9%)	7 (63.6%)	1.000
^†^Time of the initial MRI to symptom onset, d (IQR)	0.88 (1.00–3.00)	1.23 (0.52–2.75)	0.648	1.33 (1.00–2.00)	2.00 (0.56–3.50)	0.904	1.00 (0.71–2.00)	1.75 (1.00–2.00)	0.169
NIHSS, score (IQR)	0 (0–1)	1 (0–3)	**0.045**	2 (0–4)	1 (0–2.5)	0.389	0 (0–2)	0 (0–0.25)	0.103
MRI time interval, months (IQR)	7 (6–19)	11 (6–21)	0.652	7 (6–15)	6 (5–10)	0.263	6 (6–13)	14 (7–17)	**0.040**
**Vascular risk factors**									
Diabetes, n (%)	8 (29.6%)	6 (35.3%)	0.748	14 (60.9%)	3 (60.0%)	1.000	17 (33.3%)	1 (10.0%)	0.256
Hypertension, *n* (%)	23 (85.2%)	14 (82.4%)	1.000	21 (91.3%)	3 (60.0%)	0.135	42 (82.4%)	4 (40.0%)	**0.010**
Hyperlipemia, *n* (%)	4 (14.8%)	4 (23.5%)	0.690	7 (30.4%)	0 (0.00%)	0.290	9 (17.6%)	1 (10.0%)	1.000
Cardiac disease, *n* (%)	3 (11.1%)	1 (5.9%)	1.000	1 (4.3%)	0 (0.00%)	1.000	9 (16.4%)	2 (20.0%)	1.000
**Clinical index**									
CRP, mmol/L (IQR)	2.35 (1.30–6.25)	1.60 (0.70–4.40)	0.297	1.90 (0.80–7.90)	2.75 (1.18–12.13)	0.657	1.60 (0.60–3.50)	2.85 (0.50–32.30)	0.391
HbA1c,% (IQR)	6.30 (5.98–6.73)	6.20 (5.70–7.63)	0.842	7.30 (6.60–9.80)	7.50 (6.10–8.15)	0.568	6.40 (6.00–7.20)	6.00 (5.80–6.35)	0.083
Glu, mg/L (IQR)	5.72 (5.16–6.82)	4.79 (4.66–5.76)	**0.015**	6.35 (5.26–8.52)	6.43 (4.23–10.53)	0.560	5.39 (4.74–6.56)	4.85 (4.43–6.05)	0.182
Systolic pressure, mmHg (IQR)	152 (142–167)	149 (137–166)	0.595	156 (145–166)	140 (121–181)	0.210	154 (137–165)	138 (126–155)	**0.034**
Diastolic blood pressure, mmHg (IQR)	90 (76–96)	82 (74–101)	0.725	85 (76–95)	78 (73–82)	0.103	85 (76–94)	77 (70–92)	0.323
**Imaging characteristics**									
RSSIs diameter, mm (IQR)	11.70 (8.03–13.60)	8.67 (8.05–10.01)	**0.027**	13.20 (7.81–15.00)	9.76 (4.35–14.70)	0.344	9.29 (7.43–12.60)	5.80 (3.42–9.55)	**0.011**
ADC × 10^–6^, mm^2^/s (IQR)	556 (488–619)	550 (502–630)	0.956	598 (474–723)	593 (507–812)	0.684	550 (497–662)	632 (517–745)	0.102
^#^WMH volume, cm^3^(IQR)	11.03 (5.42–19.35)	10.12 (2.13–36.27)	0.833	10.63 (7.84–22.26)	33.40 (2.60–55.64)	0.564	24.52 (12.95–36.51)	10.30 (1.54–33.08)	0.064
Contact with surrounding WMH, *n* (%)	5 (18.5%)	0 (0%)	0.139	1 (4.3%)	0 (0.0%)	1.000	47 (85.5%)	2 (18.2%)	** < 0.001**

*Data are presented as median (IQR) or number (%). CRP, c-reactive protein; ADC, apparent diffusion coefficient; RSSIs, recent small subcortical infarcts; WMH, white matter hyperintensities. Bold numbers represent the significant statistical difference (*p* < 0.05) among three locations. ^†^One lesion in the basal ganglion region and six lesions in the subcortical white matter region were found by coincidence without definite time of symptom onset. ^#^Analysis of total WMH volume was performed in 162 patients (five patients had more than one RSSI at same time point) because 14 patients didn’t have baseline T2 FLAIR images.*

**TABLE 3:** Multivariate logistic regression analysis of cavitation of subcortical white matter RSSIs.

	**OR**	***P* value**	**Confidence interval**
Contact with surrounding WMH	101.760	0.003*	4.717–2,195.290
Hypertension	10.025	0.101	0.637–157.711
MRI time interval, months	0.952	0.444	0.840–1.080
RSSIs diameter, mm	1.100	0.590	0.777–1.559
WMH volume, cm^3^	0.969	0.430	0.895–1.048
HbA1c	3.252	0.131	0.702–15.057

WMH, white matter hyperintensities. Data are presented as OR (95% confidence intervals). *Values denote significant statistical difference (*p* < 0.05/3).

**TABLE 4:** Multivariate logistic regression analysis of cavitation of basal ganglion region RSSIs.

	**OR**	***P* Value**	**95% CI**
RSSIs diameter, mm	1.355	0.044	1.009–1.822
Glu	1.286	0.348	0.760–2.177
NIHSS	1.847	0.080	0.930–3.668

*RSSIs, recent small subcortical infarcts. Data are presented as OR (95% confidence intervals). No value denotes significant statistical difference (*p* < 0.05/3).*

## Discussion

In this study, we find that RSSIs in different locations have different characteristics and evolution patterns. Brainstem RSSIs are larger, accompanied by more severe symptoms, and more frequently occurring in patients with diabetes. Subcortical white matter RSSIs more frequently contact with surrounding WMH and occur in patients with a severe burden of WMH. Follow-up analyses demonstrate that RSSIs are less likely to cavitate in the basal ganglia region than in subcortical white matter. Notably, basal ganglia region RSSI cavitation is mainly associated with baseline RSSI diameter, and subcortical white matter RSSI cavitation is related to contact with WMH.

First, we assessed the baseline characteristics of RSSIs in different regions. Consistent with previous studies (Karapanayiotides et al., 2004; Subramanian et al., 2009; Palacio et al., 2014), we find that patients with brainstem RSSIs are more likely to have diabetes than patients with subcortical white matter and basal ganglia region RSSIs. Although they are frequently reported, the specific pathophysiological mechanisms linking diabetes and brainstem infarcts are still unclear. Diabetes can cause damage to the autonomic nervous system, which plays important roles in regulating cerebral blood flow (Goadsby, 2013; Purkayastha et al., 2013; Wildfong, 2016). Among blood vessels supplying the brain, the vertebrobasilar system has less sympathetic nerve supply compared with the internal carotid system (Edvinsson, 1982; Ichikawa et al., 2009; Goadsby, 2013). Therefore, the vertebrobasilar system may be more vulnerable to diabetes, and its inability to regulate blood flow may result in more RSSIs in the brainstem. In addition, we find that patients with subcortical white matter RSSIs have a severe burden of WMH, and the RSSIs also more frequently contact with surrounding WMH compared to brainstem and basal ganglia region RSSIs. These findings can be explained by the shared risk factors of RSSIs and WMH (Inzitari, 2003; Bailey et al., 2012; Traylor et al., 2016) and also evidence showing that WMH is associated with the occurrence of subcortical white matter RSSIs, independent of age and vascular risk factors, such as hypertension and diabetes (Kloppenborg et al., 2017; Pinter et al., 2018; Xu et al., 2018).

In the longitudinal analysis, we discovered that basal ganglia region RSSIs are less likely to form cavities compared to subcortical white matter RSSIs. According to neuroanatomical studies, the basal ganglia region has a higher density of microvessels (Kubíková et al., 2018), which support collateral blood flow after infarction and contribute to tissue survival, leading to less cavitation (Feekes et al., 2005). In addition, a microglial-related inflammation reaction is also minor in gray matter compared to white matter (Hart et al., 2012). In a small stroke rat model, it was demonstrated that delaying the release of inflammatory factors prevents cavity formation (Hua and Walz, 2006). Therefore, higher perfusion and less inflammation might be the reasons for less cavitation of basal ganglia RSSIs. However, because we had not measured the cerebral blood flow and inflammatory reaction in our study, this hypothesis needs to be clarified in future studies.

In addition, we also find that the factors associated with cavitation of RSSIs in different locations are distinct. RSSIs of subcortical white matter are more likely to form cavities when closely contacting with WMH, demonstrating that WMH not only contributes to RSSI formation, but also its cavitation. It is not surprising that WMH is not associated with RSSI cavitation in the basal ganglia region because WMH mainly presents in the centrum semiovale and periventricular areas (Pantoni, 2010). This discrepancy between the subcortical white matter and basal ganglia region again reveals that cavitation of RSSIs is more affected by the local surrounding environment, such as hypoperfusion and inflammation, instead of overall disease burden. Therefore, previously studies that investigated the evolution patterns of RSSIs without consideration of RSSI locations might be biased (Zhang et al., 2016; Kwon et al., 2019).

Our study had limitations. First, our study has a retrospective design in a single center and moderate sample size, which may have brought some sampling biases. We compared our study with previous RSSIs studies and find that the prevalence of hypertension and diabetes are similar (Loos et al., 2012; Pinter et al., 2018; Kwon et al., 2019) although the prevalence of hyperlipemia is lower. Our patients have higher WMH volume at baseline, which may influence RSSI development and evolution. These factors need to be considered when comparing our results with other studies. The brainstem group has the smallest sample size, and we only find a marginally significant association between the evolution of brainstem RSSIs and age (*P* = 0.050). This needs to be further verified in larger samples. Second, the follow-up time ranges from 3 months to 3 years. Nevertheless, according to previous studies, infarct evolution would be stable after 3 months (Okazaki et al., 2015), and we don’t find any association between cavitation and follow-up months in multivariable analysis. Third, we don’t analyze the relationship between cavitation and functional outcome, which needs to be investigated in future studies.

In conclusion, our study demonstrates that the occurrence of RSSIs in different locations is associated with different clinical and imaging factors. In addition, cavitation of RSSIs might be affected by local lesion features and the surrounding environment rather than general demographic and clinical factors.

## Data Availability Statement

The raw data supporting the conclusions of this article will be made available by the authors, without undue reservation, to any qualified researcher.

## Ethics Statement

The studies involving human participants were reviewed and approved by The Second Affiliated Hospital, School of Medicine, Zhejiang University. The patients/participants provided their written informed consent to participate in this study. Written informed consent was obtained from the individual(s) for the publication of any potentially identifiable images or data included in this article.

## Author Contributions

HH and RZ drafted and revised the manuscript, participated in study concept and design, conducted the statistical analyses, and analyzed and interpreted the data. MZ participated in study concept and design, data interpretation, and made a major contribution in revising the manuscript. SW, XL, and ML participated in the study design and made a contribution in revising the manuscript. YJ, PH, XY, and MZ assisted in designing the MRI sequences and imaging analysis. All authors contributed to the article and approved the submitted version.

## Conflict of Interest

The authors declare that the research was conducted in the absence of any commercial or financial relationships that could be construed as a potential conflict of interest.

## Footnotes

1 http://www.mricro.com
