# Supplementary material for: Factors Associated With the Occurrence and Evolution of Recent Small Subcortical Infarcts (RSSIs) in Different Locations
Source: Front Aging Neurosci. 2020 Aug 26;12:264. doi: 10.3389/fnagi.2020.00264 (PMC7479090; doi:10.3389/fnagi.2020.00264)
Supplement: Supplementary file 1 [file Table_1.DOCX]

**Table 1S.** Comparison of clinical data and MRI findings of RSSIs with and without follow-up MRI

|  | Without follow-up (n = 48) | With follow-up (n = 133) | P value | |
| --- | --- | --- | --- | --- |
| **Demographic characteristics** |  |  |  | |
| Age, y (IQR) | 67.5 (63.0-78.5) | 68.0 (63.5-76.5) | 0.867 | |
| Male, n (%) | 31 (64.6%) | 80 (60.2%) | 0.589 | |
| †Time of the initial MRI to symptom onset, d (IQR) | 2.00 (1.00-5.00) | 1.00 (0.97-2.00) | | **0.013** |
| **NIHSS, score (IQR)** | 1.5 (0-3.0) | 1.0 (0-2.0) | | 0.064 |
| **Vascular risk factors** |  |  | |  |
| Diabetes, n (%) | 18(37.5%) | 50(37.6%) | | 0.991 |
| Hypertension, n (%) | 41(85.4%) | 107(80.5%) | | 0.445 |
| Hyperlipemia, n (%) | 3(6.3%) | 25(18.8%) | | **0.039** |
| Cardiac disease, n (%) | 3(6.3%) | 16(12.0%) | | 0.263 |
| **Clinical index** |  |  | |  |
| CRP, mmol/L (IQR) | 1.80(0.60-5.30) | 1.90(0.80-5.05) | | 0.751 |
| HbA1c, % (IQR) | 5.90(5.60-6.95) | 6.30(6.00-7.30) | | **0.002** |
| Glu, mg/L (IQR) | 5.08(4.49-5.75) | 5.40(4.77-6.86) | | **0.005** |
| Systolic pressure, mmHg (IQR) | 152(144-166) | 152(139-166) | | 0.399 |
| Diastolic blood pressure, mmHg (IQR) | 85(75-94) | 85(76-94) | | 0.709 |
| **Imaging characteristics** |  |  | |  |
| ADC×10^-6^, mm^2^/s (IQR) | 540(469-670) | 562(496-660) | | 0.537 |
| RSSIs diameter, mm | 9.82(7.76-11.78) | 9.54(7.56-13.20) | | 0.735 |
| #Total WMH volume, cm^3^ | 15.81(7.24-36.04) | 13.57(7.45-33.26) | | 0.437 |
| Contact with surrounding WMH, n (%) | 14(29.2%) | 52(39.1%) | | 0.220 |
| **Location** |  |  | |  |
| Brainstem, n (%) | 11(22.9%) | 28(21.1%) | |  |
| Basal ganglia region, n (%) | 27(56.3%) | 61(45.9%) | | 0.271 |
| Subcortical white matter, n (%) | 10(20.8%) | 44(33.1%) | |  |

Data are presented as median (IQR) or number (%)

CRP: C-reactive protein, ADC: apparent diffusion coefficient, RSSIs: recent small subcortical infarcts, WMH: white matte hyperintensities

Bold numbers represented the significant statistical difference (p<0.05) between groups

† Six lesions without follow-up MRI and 7 lesions with follow-up MRI were found by coincidence without definite time of symptom onset.

# Analysis of total WMH volume were performed in 41 patients without follow-up (7 patients without follow-up didn’t have baseline T2FLAIR images) and in 121 patients with follow-up (7 patients with follow-up baseline T2FLAIR images, and 5 patients had two RSSIs at the same time point)
